# Analysis of cyclohexane, cyclopentane, and benzene conformations in ligands for PDB X-ray structures using the Hill-Reilly approach

**DOI:** 10.1186/s13321-026-01154-0

**Published:** 2026-01-10

**Authors:** Gabriela Bučeková, Viktoriia Doshchenko, Tomáš Svoboda, Jana Porubská, Aliaksei Chareshneu, Tomáš Raček, Vladimír Horský, Radka Svobodová, Ondřej Schindler

**Affiliations:** 1 National Centre for Biomolecular Research, Masaryk University, Kamenice 753/5, 625 00 Brno, Czech Republic; 2 CEITEC - Central European Institute of Technology, Masaryk University, Kamenice 753/5, 625 00 Brno, Czech Republic

**Keywords:** Cyclohexane, Cyclopentane, Benzene, Ring conformation, Ligand, X-ray crystallography, Protein Data Bank, Hill-Reilly approach

## Abstract

Protein structural data are highly valuable for research, and many significant results have been published on their basis. An important part of protein structures is their ligands. The conformation of rings in ligands is crucial for the ligands’ scaffold and shape and, therefore, for interactions with their surroundings and the subsequent biological function. For this reason, we developed a workflow to detect conformations of cyclohexane, cyclopentane, and benzene rings. The workflow can process rings originating from ligands, which are parts of experimental protein structures deposited in the Protein Data Bank and determined by X-ray crystallography. This fully automatic workflow utilises the Hill-Reilly approach to calculate puckering angles that quantitatively describe ring conformation. The reproducibility of the workflow is guaranteed by storing datasets within Onedata, which enables automatic dataset retrieval and the workflow execution. We analysed 128 012 ring structures originating from 25 479 different ligands. We found that cyclohexane ring structures include more than 22 % of unfavourable conformations, cyclopentane ring structures about 5 % and benzene ring structures only 0.01 %. We discovered that energetically unfavourable ring structures can occur in cyclohexane and cyclopentane ligands for proper chemical reasons. Their examination can help us to understand the binding of these ligands, which can be helpful for pharmacology, chemoinformatics, etc. On the other hand, energetically unfavourable ring conformations are often caused by model quality issues. Therefore, their occurrence should motivate researchers to inspect the quality of the protein model and also the ring’s fit into experimental data.

**Scientific Contribution:** Our analysis uncovers a conformational behaviour of cyclohexane, cyclopentane, and benzene rings, occurring in ligands, which are parts of experimental protein structures deposited in the PDB. This paper’s other substantial contribution is presenting the first successful application of the Hill-Reilly approach for cyclohexane, cyclopentane, and benzene rings. Moreover, we provide Hill-Reilly parameters for these ring conformations, which can be used in other analyses.

**Graphical Abstract:**

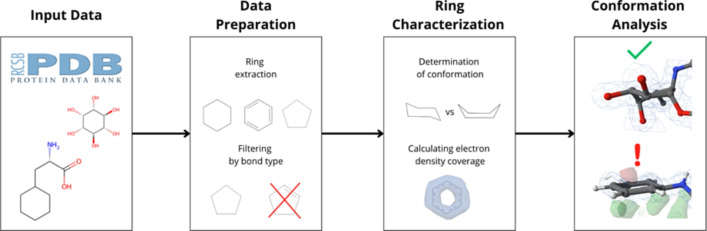

## Introduction

Experimentally determined protein structures, freely available in the Protein Data Bank (PDB) [1], form a highly valuable dataset. Ligands are often present in these structures, and their interactions with proteins are an object of intensive research in fields such as drug design [2, 3], enzymology [4], or glycoinformatics [5, 6].

The conformation of a ligand is crucial for its interactions and subsequent biological function. Therefore, examining the conformation of bound ligands in protein structures in the PDB is important. Moreover, determining a ligand’s conformation is essential for describing its binding mode and the binding site geometry or searching for other potential ligands suitable for a specific binding [7–9].

One of the key parts influencing ligand conformation is rings, which strongly influence the formation of the ligand’s scaffold and shape.

Most rings can form several conformations that differ by their potential energy and, consequently, their stability. It was theoretically determined which conformations common rings can adopt [10–12]. However, we have much less information about ring conformations in experimentally acquired structures. Specifically, the ring conformations in experimental structures are usually analysed only for heterocycles, such as those found in saccharides [5, 13]. A study aiming to determine the conformation of common homocycles (i.e., cyclohexane, cyclopentane, benzene) from experimentally determined protein structures has not yet been published despite these rings being abundantly found in ligands. In our work, we focus on these homocyclic rings and discern the conformation they adopt in ligands from the PDB.

We developed a bioinformatics workflow to detect the conformation of a ring. The core part of the workflow is based on the newest and well-established Hill-Reilly approach [14]. This approach defines the conformation of a general puckered ring based on the angles between planes passing through the selected atoms of the analysed ring [15]. It extends the common Cremer-Pople approach, which provides a quantitative method for describing three-dimensional ring conformations [16]. The Hill-Reilly approach is particularly advantageous for analysing small ring systems, where traditional descriptors can lack precision [14].

By applying our workflow, we can answer the following questions:

- In what conformations do cyclohexane, cyclopentane, and benzene rings occur in the experimental data? And with what frequency?
- Do the experimental data support the unfavourable ring conformations?
- Can the rings also validly occur in energetically unfavourable conformations?

Answers to these questions are essential because the unfavourable conformations confirmed by experimental data are very interesting in investigating the ligand binding modes. On the other hand, detecting unfavourable conformations that lack experimental data support is important in validating the ligand structure. Rings with unfavourable conformations not supported by the underlying measured data pose a potential quality issue in the structure model.

We analysed all the cyclohexane, cyclopentane, and benzene rings in the PDB structures to get data that would serve as a foundation for articulating responses to our questions. We have included only structures obtained using X-ray crystallography in the analysis. This decision allowed us to use electron density in two ways to examine whether experimental data support the conformation of a ring. Firstly, we checked whether the structure has sufficient resolution (i.e., better than 2 Å) to provide enough data to determine the ring conformation. Secondly, we determined if all the ring atoms are covered by electron density. Using only structures determined by X-ray crystallography is not a significant limitation since more than 80 % of PDB structures were determined by this established experimental method (https://www.rcsb.org/stats/summary).

Both the analysis and the workflow are beneficial contributions of this work to the scientific community. Notably, the workflow can be applied to other homocycles and 5- to 6-membered heterocycles with a single heteroatom. In general, after developing an algorithm to consistently determine the first and second atoms of a ring, the workflow can be applied to any ring. We provide its ready-to-run executable version as well as its source code.

## Methods

### Analysed rings

**Fig. 1:**
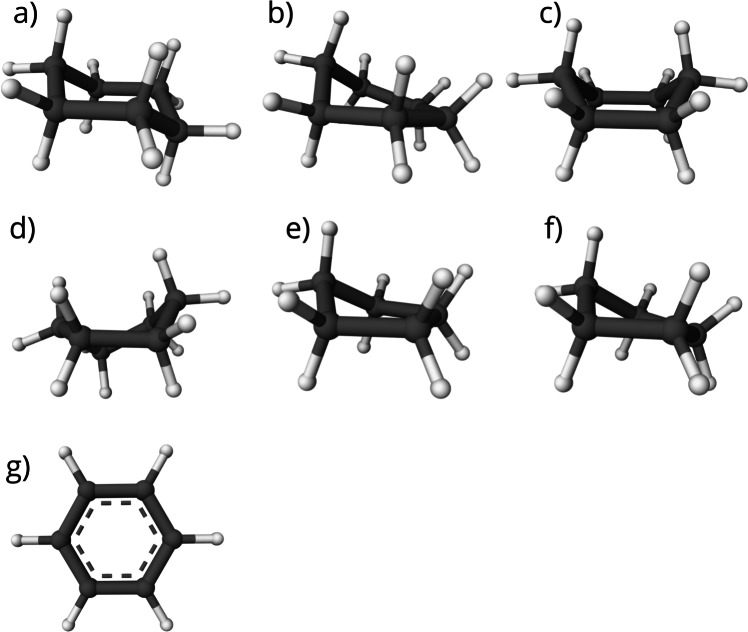
Conformations of analysed ring types: Cyclohexane: **a** chair, **b** half-chair, **c** boat, **d** twist-boat. Cyclopentane: **e** envelope, **f** half-chair. Benzene: **g** flat. The Mol* visualisation software [17] was used to prepare the figure

Our analysis focuses on three types of rings: cyclohexane, cyclopentane, and benzene. Conformations of these ring types are described in [10–12] and depicted in Fig. 1.

The conformation of a molecule is responsible for its total energy. Bonds in a ring have limited rotatability, leading to various strains in a molecule. Cyclohexane can take on one of two low-energy conformations: the chair and the twist-boat (as shown in Fig. 1a and d). The chair conformation is the more energetically favourable and stable of the two. Cyclohexane also has two unstable transition states: the half-chair and the boat (as shown in Fig. 1b and c). Cyclohexane is found in a transition state only during the change between low-energy conformations [11]. The chair conformation has carbons 1 and 4 situated above and below the plane, determined by the remaining carbon atoms. The boat conformation is obtained by having carbons 1 and 4 both above or below the plane. The twist-boat conformation is obtained from the boat conformation by twisting two parallel coplanar carbon-carbon bonds against each other. The half-chair conformation has four coplanar neighbouring carbons.

In the case of cyclopentanes, there are two known low-energy conformations: the envelope and the half-chair (as shown in Fig. 1e and f). The energy of the two conformations is similar, and cyclopentane continuously pseudorotates between them [18]. The envelope conformation has four carbons in a single plane, with the fifth atom being lifted. The half-chair conformation is obtained by slight twisting of the envelope conformer, causing only three carbon atoms to remain coplanar, while carbons 3 and 5 are at different heights above the plane determined by the remaining carbons [10].

Bonded substituents strongly influence the ring conformation [19, 20]. Thus, substituted cyclohexanes and cyclopentanes can adopt conformations considered energetically unfavourable for unsubstituted rings. Both ring types may, in rare cases, adopt a flat conformation when substituted, where all atoms approximately lie in a plane.

On the other hand, benzene naturally occurs only in the flat conformation (Fig. 1g) due to delocalised electrons forming two p-orbitals below and above the ring and, along with $\sigma$ electrons, forcing the benzene to adopt the regular hexagon shape with bond angles being 120$^\circ$ [12].

### Calculation of Hill-Reilly parameters

**Fig. 2:**
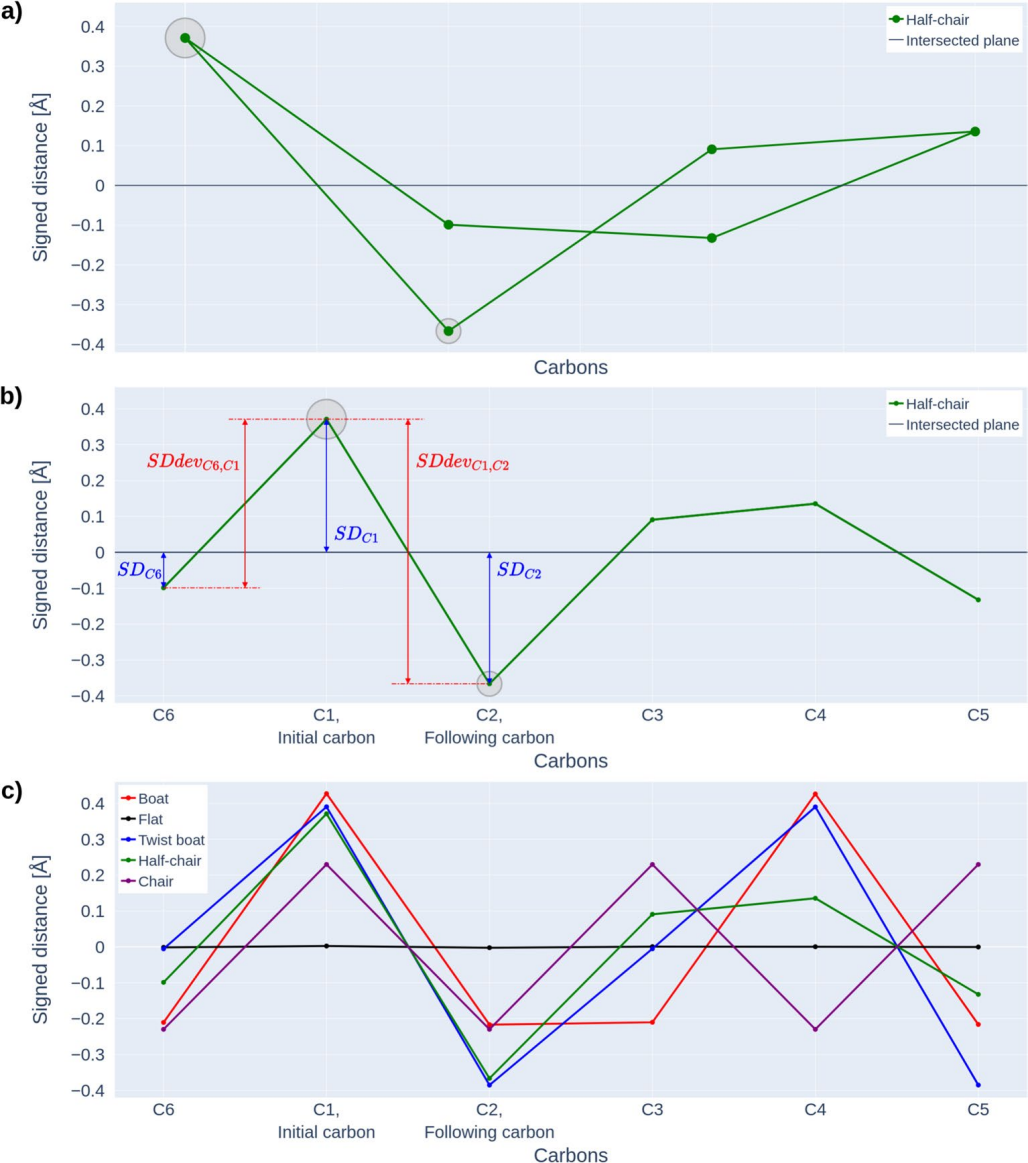
Illustrative plots for the approach of choosing the initial and following carbons for calculating the Hill-Reilly parameters. **a** Plot of sd of carbons of the cyclohexane half-chair conformation projected in 2D to the best-fit plane. A large and small grey circle highlights the expected initial and following carbons. **b** Plot of sd of carbons of the cyclohexane half-chair conformation. The plot demonstrates the meaning of *SDdev* and the process of finding the initial and following carbons. A large and small grey circle highlights the expected initial and following carbons. **c** Plot of sd of carbons of the cyclohexane conformations. The determined initial and following carbons provided the ordering of the carbons in the plot

The Hill-Reilly parameters are defined as the angles between planes passing through specific ring atoms [14]. The Hill-Reilly approach has never been applied to the conformation analysis of cyclohexane, cyclopentane, or benzene rings. For this reason, we calculated these parameters to use them in our analysis in these three steps:

1. **Preparation of optimal 3D structures of ring conformations:** We prepared 3D structures of low-energy conformations of studied rings in Jmol [21]. Afterwards, we optimised these 3D structures using ORCA [22]. Less stable conformations were prepared directly in ORCA from their low-energy equivalents using constrained and saddlepoint optimisations. Every structure was then optimised using ORCA by the DFT method at the PBE/def2-TZVP level, employing D4 dispersion corrections and the SMD solvation model. We selected this setup based on recommendations from the paper by Bursch et al. [23]. These optimised structures are available in a persistent public share (https://doi.org/10.58074/6krq-v784).
2. **Determination of the initial and following atoms for the Hill-Reilly approach:** Calculating Hill-Reilly parameters requires identifying an initial atom (to start the sequence) and a following atom (to define the direction). The Hill-Reilly approach has always been applied to heterocycles (i.e., rings containing atoms other than carbons), where the initial and following atoms are determined by the presence and position of the heteroatom in a ring. Because the rings we studied do not contain any heteroatoms, we had to apply a different approach to determine the initial and following atoms. Our approach begins by defining a best-fit plane for the ring carbons, calculated to minimise the total distance from the plane to all carbons (see an example in Fig. 2a). It then calculated the signed distance (SD) of each carbon from this plane, assigning positive values to atoms located above the plane and negative values to those below it. See Fig. 2b, which depicts a plot of carbon atoms’ signed distances for the structure of the cyclohexane half-chair conformation. We defined the deviation for two neighbouring carbons, *x* and $x+1$, as
  1 $$\begin{aligned} SDdev_{Cx,Cx+1} = | SD_{Cx} - SD_{Cx+1}|. \end{aligned}$$
  We calculated a sum of SD deviations from both their neighbours for all ring carbons as
  2 $$\begin{aligned} SDsum_{Cx} = SDdev_{Cx,Cx-1} + SDdev_{Cx,Cx+1}. \end{aligned}$$
  The carbon with the highest value of *SDsum* was determined to be the initial atom (C1) for the Hill-Reilly approach. The neighbour of C1 with the higher *SDdev* value was designated as the following atom (C2). This procedure is illustrated in Fig. 2b. Signed distances of carbons ordered by the initial and following atoms for all cyclohexane conformations are shown in Fig. 2c.
3. **Calculation of Hill-Reilly parameters:** After determining the initial and following carbon atoms, we calculated the Hill-Reilly parameters for the studied ring conformations using modified scripts from Hill et al. [14]. The Hill-Reilly parameters for two mirror-inverted rings differ only in their sign. Because we did not distinguish mirror-inverted rings in our analysis, we multiplied all parameters by -1 if the last parameter was negative. The obtained parameters are available in Table 1.

### Workflow of the analysis

**Fig. 3:**
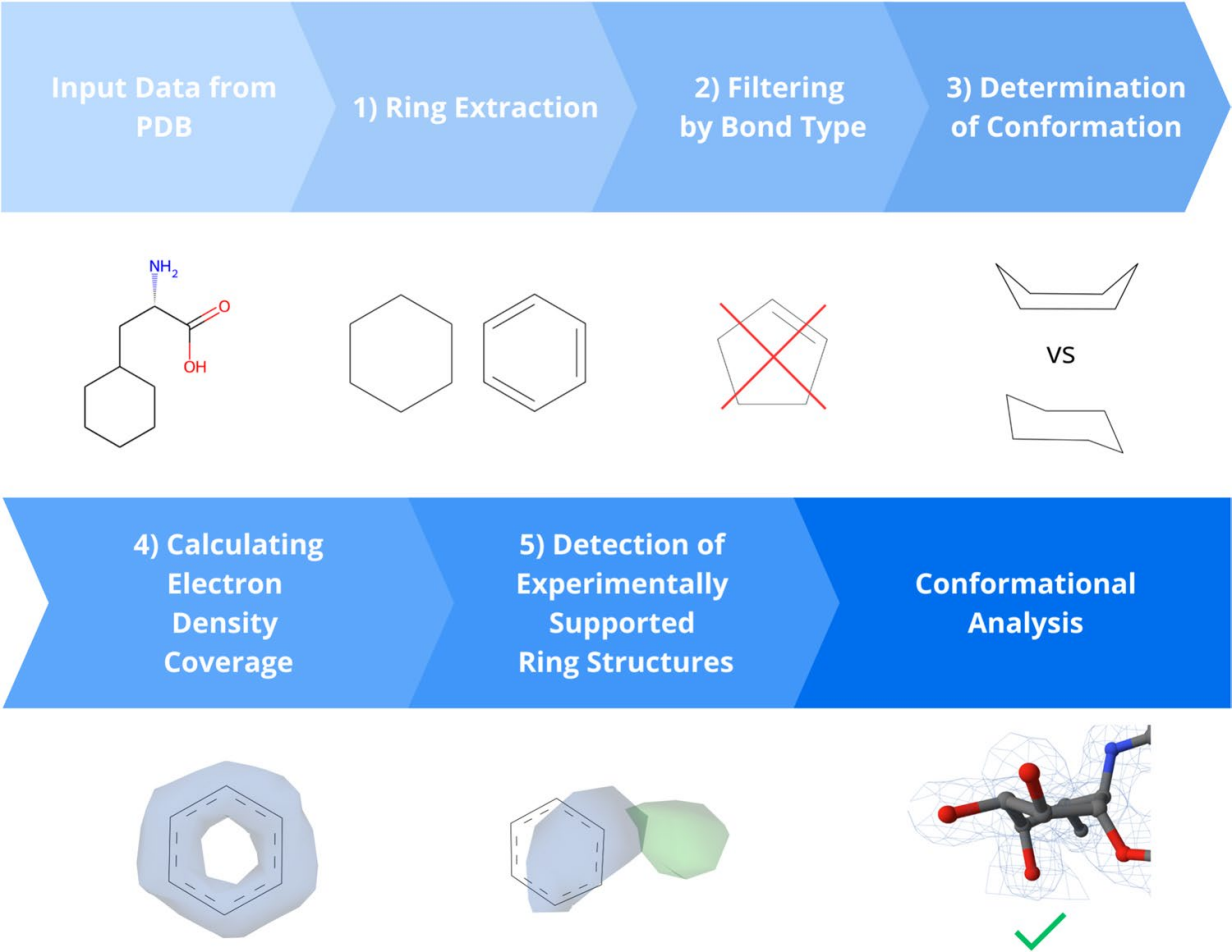
Schematic representation of the conformational analysis workflow

We used a workflow depicted in Fig. 3 and described below for the conformational analysis of cyclohexane, cyclopentane, and benzene rings (i.e., target ring types of the analysis). The workflow is fully automatic and consists of five steps: ring extraction, filtering by bond type, determination of conformation, calculation of electron density coverage, and detection of experimentally supported ring structures. The reproducibility of the workflow is guaranteed by storing datasets within the Onedata data management system [24], which enables automatic dataset retrieval and the workflow execution.

#### Ring extraction

As the first step, we selected ligands from the Chemical Component Dictionary(CCD) [25] containing at least one cyclohexane, cyclopentane, or benzene ring. Subsequently, we extracted the rings with 5 or 6 carbons from all instances of these ligands across the whole PDB via the PatternQuery tool [26]. As a result, we obtained each extracted ring as a separate PDB file [27].

#### Filtering by bond type

PatternQuery uses its own query language, which, unfortunately, cannot distinguish between different bond types. Hence, our extracted rings from PDB required an additional filtration step to remove rings with unwanted types of bonds. For this step, we again used the CCD to get the information about bond types by mapping the atom names of our extracted ring structures in PDB files to corresponding atom names in the CCD. Using this classification, we divided the remaining rings into cyclohexanes, cyclopentanes and benzenes.

#### Determination of conformation

We determined the conformation of the analysed rings using the Hill-Reilly algorithm [14]. For each ring, we first selected the initial and following atoms as described in "Description of the datasets" section and calculated the Hill-Reilly parameters. As in "Description of the datasets" section, we multiplied these parameters by -1 if the last parameter was negative. Then, we calculated Euclidean distances between the Hill-Reilly parameters of the analysed ring and the parameters of each conformation of the relevant ring type. Finally, the conformation with the shortest distance was assigned to the analysed ring. For benzene, restricted to a single defined conformation, we expanded the comparison set to include cyclohexane conformations (chair, half-chair, boat, and twist-boat) utilising their standard parameters.

#### Calculating electron density coverage

The next step of the analysis determined whether all atoms of the analysed rings are fully covered by electron density. We obtained electron density maps from the PDB database.

We computed the electron density in two steps. First, the algorithm determined the electron density value for the coordinate of the centre of an individual atom via trilinear interpolation of the eight nearest nodes on the electron density map. Then, the obtained interpolated electron density value was compared with the threshold of the electron density isosurface. This threshold was set to 1.5 $\sigma$ in our case [28], as this value is one of the most commonly used values in molecular viewers [17, 29]. If the obtained electron density value was higher than the threshold, the electron density covered the given atom. Otherwise, it was considered not covered. This process was repeated for each atom in a ring. Finally, we identified rings where at least one atom lacked coverage. We used the Python library Gemmi [30] to work with electron density data.

#### Detection of experimentally supported ring structures

In this step, we detected ring structures which are supported by experimental data, i.e., they have been refined to a higher resolution than 2 Å, and all their atoms are covered by electron density. We considered only structures with a resolution of 2 Å or better since electron density maps with lower resolution tend to lack the information content needed to determine ring conformation confidently [31, 32]. Furthermore, all ring atoms should be covered by electron density to correctly determine the location of constituent atoms and the possible conformation of the ring [33]. Therefore, only fully covered ring structures were considered as supported by experimental data.

To implement this, we aggregated the results of the previous steps into a single file in Comma-separated values (CSV) format. Then, we enriched the results with the resolution value of the structure of each target ring. The resolution values were acquired from the ValTrendsDB [34].

### Limitations

During the analysis, we encountered a few limitations. Firstly, some target benzenes that share one or more edges in polycyclic molecules (e.g., naphthalene and anthracene) are incorrectly identified as cyclohexadienes and omitted from the analysis. This limitation stems from the workflow’s current implementation of target ring detection. Secondly, as described in "Ring extraction" section, our workflow works with the PDB format. Therefore, only structures that can be written in the PDB format are included in our analysis. Thirdly, the cyclopentadiene ring of the ligand with CCD ID ORS is considered as cyclopentane by our workflow, because the ring of this ligand is incorrectly recorded as cyclopentane in CCD in the mmCIF file.

## Results and discussion

### Hill-Reilly parameters for ring conformations

The first result in this publication consists of computed parameters for the Hill-Reilly algorithm for cyclohexane, cyclopentane, and benzene rings (see Table 1). These parameters have not yet been published. Moreover, they were successfully used in our workflow.

**Table 1:** Calculated Hill-Reilly parameters for optimised ring conformations

		$\theta _1$	$\theta _2$	$\theta _3$
Cyclohexane	Chair	32.2	32.2	32.2
	Half-chair	16.1	−20.9	53.0
	Boat	−30.8	−31.7	64.4
	Twist-boat	−0.7	−57.4	58.2
	Flat	0.1	−0.0	0.4
Cyclopentane	Half-chair	−33.2	36.2	
	Envelope	−25.9	40.7	
	Flat	−0.2	0.2	
Benzene	Flat	0.0	0.0	0.0

### Description of the datasets

The analysis was performed on data acquired on 30th April 2025 from the PDB database. We divided the data into three datasets. The cyclohexane dataset contains 7,528 rings from 1,694 ligands; the cyclopentane dataset comprises 5,100 rings from 882 ligands; and the benzene dataset includes 115,384 rings from 22,903 ligands. In total, we analysed 128 012 rings. The sum of ligand counts over the three ring types is 25,479, but ligands that contain more than one of these ring types are counted once per ring type. Detailed information about the datasets is summarised in Table 2.

**Table 2:** Representation of the analysed rings in the PDB

	Number of distinct ligands in the CCD	Number of ligand instances in the PDB	Number of analysed rings
Cyclohexane	1 694	3 482	7 528
Cyclopentane	882	2 259	5 100
Benzene	22 903	40 254	115 384
Total	25 479	45 995	128 012

**Table 3:** Occurrence of individual conformations in the datasets of cyclohexane, cyclopentane, and benzene rings

Type of ring	Conformation	All rings		Rings supported by exp. data	
		Number	Occurrence [%]	Number	Occurrence [%]
Cyclohexane	Chair	5 852	77.74	1 345	17.87
	Half-chair	549	7.29	138	1.83
	Boat	541	7.19	30	0.40
	Twist-boat	513	6.81	34	0.45
	Flat	73	0.97	13	0.17
	Energetically favourable	5 852	77.74	1 345	17.87
	Energetically unfavourable	1 676	22.26	215	2.86
	Total	7 528	100.00	1 560	20.72
Cyclopentane	Half-chair	2 919	52.24	723	14.18
	Envelope	1 917	37.59	389	7.63
	Flat	264	5.18	36	0.71
	Energetically favourable	4 836	94.82	1 112	21.80
	Energetically unfavourable	264	5.18	36	0.71
	Total	5 100	100.00	1 148	22.51
Benzene	Flat	115 326	99.95	29 842	25.86
	Chair	22	0.02	12	0.01
	Half-chair	31	0.03	3	0.00
	Boat	4	0.00	1	0.00
	Twist-boat	1	0.00	0	0.00
	Energetically favourable	115 326	99.95	29 842	25.86
	Energetically unfavourable	58	0.05	16	0.01
	Total	115 384	100.00	29 858	25.88

The structures, which are supported by experimental data, have a resolution of at least 2 Å, and all ring atoms are covered by electron density

### Analysis of the cyclohexane dataset

Our analysis of the cyclohexane dataset (see Table 2) revealed that 1 694 unique ligands in the CCD contain at least one cyclohexane ring. 7 528 instances of this ring can be found in 3 482 entries in the PDB. Cyclohexane rings in the dataset occur in five conformations: one stable conformation (chair conformation) and four energetically unfavourable conformations (boat, half-chair, twist-boat, and flat). The majority (77.74 %) occurs in the chair conformation, and the remaining 22.26 % (i.e., 1 676 ring structures) are in energetically unfavourable conformations (see Table 3). Specifically, the boat conformation was observed in 7.19 % of the dataset. 14.1 % of cyclohexane rings were in the half-chair or twist-boat conformation, which are transition states [35, 36], and less than 1 % of the rings were in the flat conformation.

We directed our attention to the energetically unfavourable ring structures having sufficient experimental data to ascertain their conformation (i.e., the resolution is equal to or higher than 2 Å, and all atoms are covered by electron density). Our dataset contains 215 such rings (see Table 3). We examined these ring structures and found that many do not fit the measured electron density. Specifically, they are surrounded by red and green blobs in PDB-style electron density maps [33]. These blobs indicate differences between the computed electron density from the structure model and the measured electron density, which in practice means that the modelled structure of a ligand does not represent the underlying measured experimental data well [33]. Also, some rings contain wrong annotation of the ring, such as incorrect bond assignments, leading to misidentification.

All 13 experimentally supported flat ring conformations in our dataset contain structure fit or annotation issues. The ligand with CCD ID NHE is among the most frequently observed ligands in this group, appearing in the PDB entries 1oks, 1zhh, and 3oro [37–39]. NHE is a commonly used buffering agent. Since in PDB-REDO [40], which is a database of optimised structures from the PDB, the cyclohexane rings of NHE ligands are found in a chair conformation, we can conclude that the problem is caused by the use of incorrect restraints during model refinement. Another example of a flat cyclohexane with a problematic structure fit caused by the usage of wrong restraints during model refining is a ligand with CCD ID 9NW, shown as a case study in "Poorly fitted flat conformation of cyclohexane in PDB entry 6tm9" section.

However, we also found cases where the energetically unfavourable conformations, such as boat, half-chair, or twist-boat, have a structural reason. Most of the cyclohexanes found are held in an energetically unfavourable conformation by their multiple substituents bound to the protein and their stereochemistry. These effects are evident in the case of rings in ligands with CCD IDs 3U3 (4 instances of rings found to be supported by experimental data), HZB (1 instance), OJ6 (1 instance), VEE (1 instance), and KHG (1 instance). Multiphosphorylated cyclohexanes, such as the rings in ligands with CCD IDs IHP (7 instances) and 4IP (2 instances), are also in an energetically unfavourable conformation for the same reason. Energetically unfavourable conformation (e.g., half-chair) can also be caused by cyclohexane substituents forming another ring, as we can see, e.g., in a ligand with CCD ID PID (72 instances). A minority group consists of ligands whose rings are held in the half-chair conformation by two double-bonded oxygens. However, for these rings, the half-chair conformation is not energetically unfavourable due to sp^2^ hybridisation of oxygen-bonded carbons, the keto-enol equilibrium, and associated resonances. This phenomenon is observed in ligands with CCD IDs CXO (2 instances) and M69 (1 instance). A ring in the half-chair conformation in ligand 3U3 is discussed in "Proper half-chair conformation of a cyclohexane in PDB entry 4wsj" section.

### Analysis of the cyclopentane dataset

The cyclopentane dataset (see Table 2) reveals 882 unique ligands in the CCD with at least one cyclopentane ring in their molecular structure. In the PDB, we found 5 100 instances of the ring in 2 259 entries. The cyclopentane rings in the dataset were found to have one of three conformations: two stable ones (half-chair and envelope) and one energetically unfavourable (flat). Most of the rings were in stable conformations, and only about 5.18 % of cyclopentane rings (i.e., 264 rings) in the dataset were in flat conformation (see Table 3).

Similarly to cyclohexanes, we focused on the energetically unfavourable (flat) conformations. We found that only 36 have sufficient experimental data to ascertain their conformation, i.e., their resolution is 2 Å or better, and all carbons in the cyclopentane ring are covered by electron density. Therefore, the remaining 228 flat cyclopentane rings seem to have been assigned their conformation without adequate proof supporting their existence.

As a last step of the cyclopentane analysis, we examined the experimentally supported flat ring structures in detail. We found that many of them do not fit the measured electron density, as documented by the occurrence of red and green blobs in their PDB-style electron density maps. An example of a cyclopentane in the flat conformation, which does not fit in the electron density and should be in a more energetically favourable conformation, is discussed in "Poorly fitted flat conformation of cyclopentane in PDB entry 4r91" section.

On the other hand, we also found ring structures, which are in the flat conformation for proper chemical reasons. For example, in the ligand with CCD ID FGO (10 instances), the flat conformation is caused by its substituents forming another five-membered ring. In ligands with CCD ID K9R (2 instances), the flat conformation is not unfavourable. Their flat conformation is held by two double-bonded oxygens, sp^2^ hybridisation of oxygen-bonded carbons, the keto-enol equilibrium, and associated resonances. One instance of a flat cyclopentane ring in ligand K9R is discussed in "Proper flat conformation of a cyclopentane in PDB entry7zxz" section.

### Analysis of the benzene dataset

The benzene dataset includes 22 903 unique ligands from the CCD (as shown in Table 2). These ligands contain 115 384 instances of the benzene ring present in 40 254 entries in the PDB. Most benzene structures (i.e., 99.95 %) adopt the energetically favourable flat conformation. Only 58 benzene rings are in energetically unfavourable conformations akin to cyclohexane’s conformations (i.e., chair, half-chair, boat, and twist-boat), as is documented in Table 3.

From the unfavourable benzene structures, 16 samples meet the criteria for further analysis (high resolution and all atoms covered by electron density). Therefore, we examined them in detail. We found some model quality issues in all of them. Mostly, they had a poor fit to the electron density. Nevertheless, there are also other issues. For example, the ligand with CCD ID 2CH should have a benzene ring according to CCD, but it has chair conformation in the protein 8ags [41]. Since the chair conformation is clearly supported by electron density, this is an annotation error. "Poorly fitted chair conformation of a benzene in PDB entry 3ilz" section shows one example of a benzene in a non-planar conformation (specifically, the chair conformation) and its problematic fit to the electron density.

### Case studies

We selected five representative structures to illustrate some of the analysed rings found in energetically unfavourable conformations in the PDB.

As part of the analysis, we examined the corresponding electron density maps. Generally, green and red blobs in an electron density map show a discrepancy between the electron density computed from the structure and the electron density measured during an X-ray crystallography experiment. Green blobs show places with too few electrons modeled at that position. Red blobs indicate parts of the structure model that should not be present according to the experimental data [33].

A common reason for energetically unfavourable ring conformations is the use of wrong restraints during model refinement. These restraints include bond lengths, bond angles, planes, and chirality [42]. Restraints play a crucial role in refining macromolecule and ligand structures because they represent the right chemical description of the ligand. The use of wrong restraints during model refinement is the reason for the incorrect conformation of the cyclohexane in the following Case Study.

#### Poorly fitted flat conformation of cyclohexane in PDB entry 6tm9

The structure of PDB entry 6tm9 [43], published in 2020, includes a ligand with CCD ID 9NW. This ligand has been investigated as a potential inhibitor of antibiotic-resistant metallo-$\beta$-lactamases. The structure was determined at a high resolution of 1.07 Å.

**Fig. 4:**
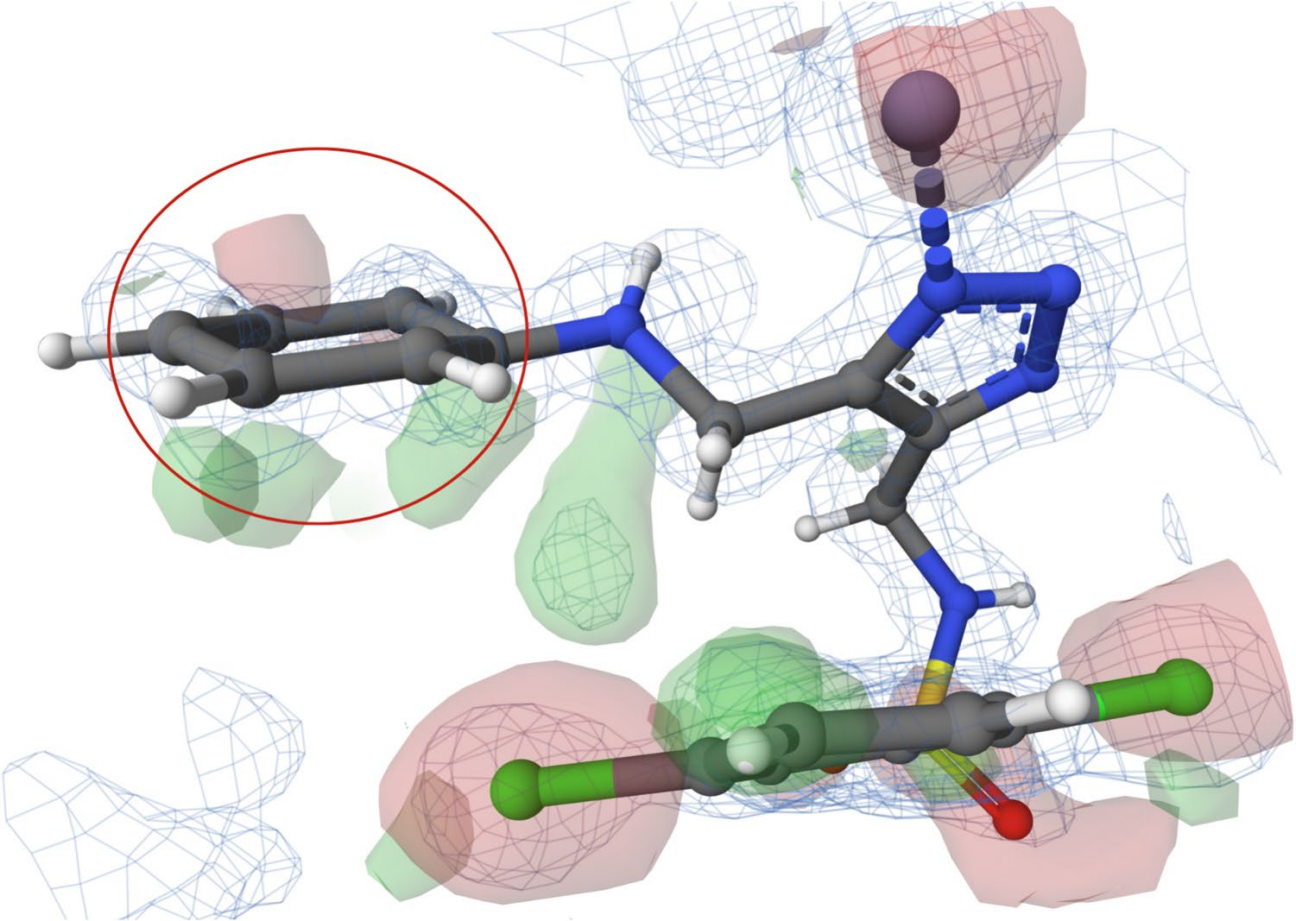
The ligand 9NW (chain B, residue number 401) from PDB entry 6tm9 [43] with cyclohexane ring in flat conformation (highlighted in a red circle). Note the cyclohexane hydrogens, which are assigned in the same way as in benzene. The ligand is surrounded by electron density, which is visualised using a PDB-style electron density map [33]. The Mol* visualisation software [17] was used to prepare the figure

The ligand, which contains a flat cyclohexane, is situated in the substrate-binding site of the macromolecular structure (see Fig. 4). Two atoms are slightly out of plane, indicating a chair conformation, but not enough to assign this conformation. All atoms of the ligand are covered by electron density. During the analysis, we identified problematic areas concerning the fit of the ligand molecule to the electron density measured during the X-ray experiment. The problematic areas are visualised as green and red blobs near the analysed ring in the PDB-style electron density map. Consequently, the electron density data do not support the existence of a planar conformation for this cyclohexane instance.

Additionally, the cyclohexane is not substituted, and its atoms have only 2 interactions with a water molecule (see its binding interactions here: https://www.ebi.ac.uk/pdbe/entry/pdb/6tm9/bound/9NW). Therefore, there is no structural reason for its atoms to adopt an energetically unfavourable flat conformation.

It is evident that this flat conformation of cyclohexane is caused by wrong restraints in model refinement, because in the PDB-REDO database, this cyclohexane is found in a chair conformation. This fact is also supported by the cyclohexane hydrogens, which are assigned in the same way as in benzene.

#### Proper half-chair conformation of a cyclohexane in PDB entry 4wsj

The structure of PDB entry 4wsj [44], published in 2014, includes a ligand with CCD ID 3U3. The ligand is a selective, irreversible inhibitor and labelling probe for GH29 $\alpha$-L-fucosidase enzyme. The structure was determined at a resolution of 1.64 Å.

**Fig. 5:**
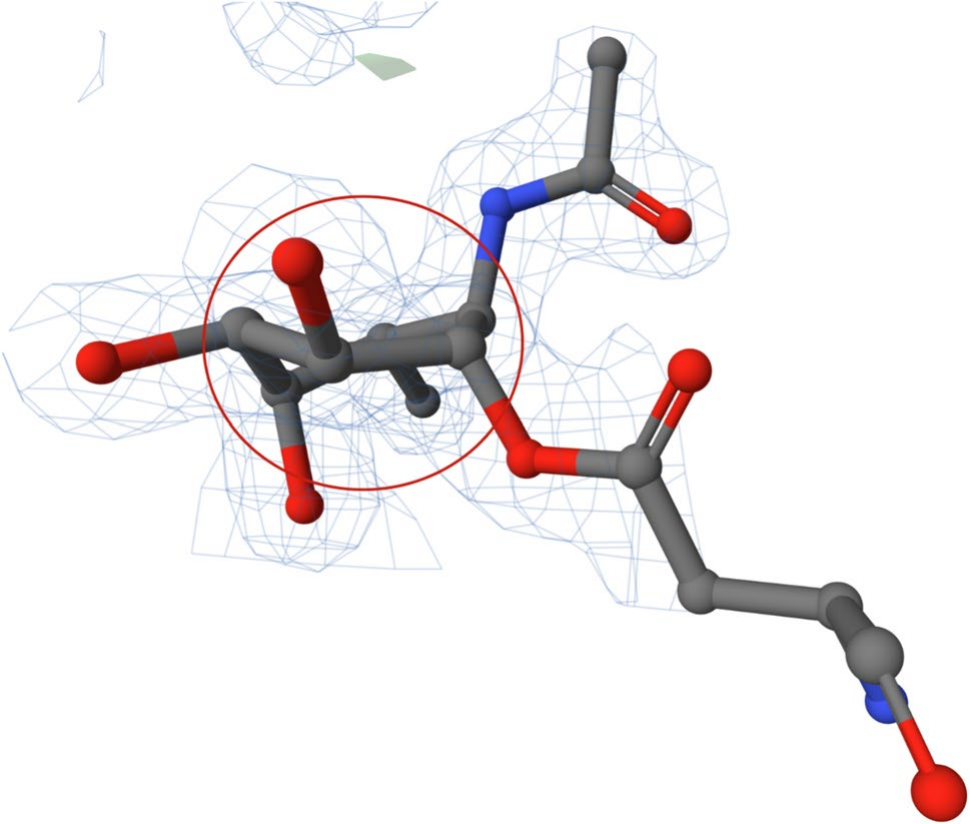
The ligand 3U3 (chain G, residue number 503) from PDB entry 4wsj [44] with cyclohexane ring in half-chair conformation (highlighted in a red circle). The ligand is surrounded by electron density, which is visualised using a PDB-style electron density map [33]. The Mol* visualisation software [17] was used to prepare the figure

The cyclohexane ring is observed in the half-chair conformation (see Fig. 5), which is the transition state for this ring type. All atoms of the ligand are surrounded by electron density. There are no green or red blobs of electron density around the ligand to indicate regions of electron deficiency or excess.

**Fig. 6:**
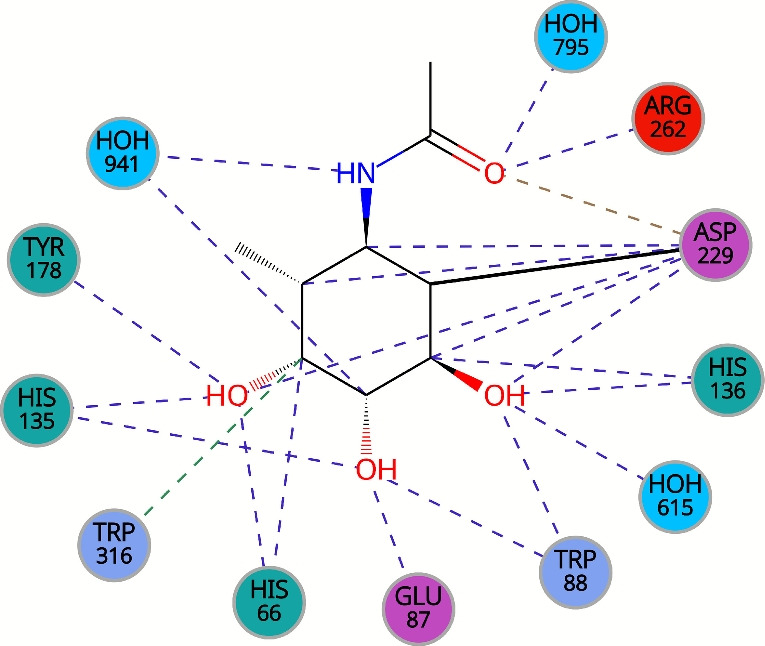
Environment details of ligand 3U3 from PDB entry 4wsj [44]. The figure is available at https://www.ebi.ac.uk/pdbe/entry/pdb/4WSJ/bound/3U3. Note that the figure uses author-provided chain IDs. Therefore the indexing of the residues is different than in the text

The ligand is covalently bound to the aspartic acid residue (ASP 195) of $\alpha$-L-fucosidase, preventing the function of this enzyme. Moreover, the substituents bound to the cyclohexane ring in the ligand interact with the surrounding residues, as seen in Fig. 6. These specific interactions, combined with the excellent fit to the electron density map, confirm the validity of the half-chair conformation.

#### Poorly fitted flat conformation of cyclopentane in PDB entry 4r91

The structure of PDB entry 4r91 [45], published in 2014, includes a ligand with CCD ID 3KT. This ligand has been investigated as a prospective inhibitor of the aspartyl protease BACE1 and, thus, as a potential treatment for Alzheimer’s disease. The structure was determined at a resolution of 1.58 Å.

**Fig. 7:**
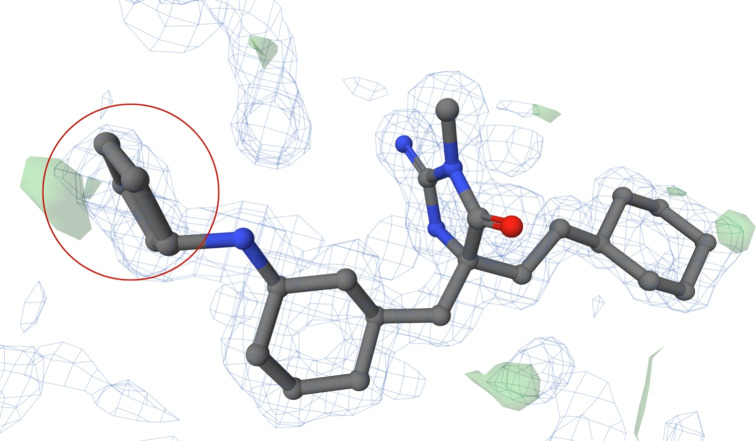
The ligand 3KT (chain D, residue number 502) from PDB entry 4r91 [45] with cyclopentane ring in flat conformation (highlighted in a red circle). Both cyclohexane rings of the ligand are in energetically favourable chair conformation. The ligand is surrounded by electron density visualised using a PDB-style electron density map [33]. The Mol* visualisation software [17] was used to prepare the figure

The analysed cyclopentane ring is observed in a flat conformation in the ligand 3KT (see Fig. 7). The entire ligand is well-defined in electron density, including the cyclopentane ring. Furthermore, both cyclohexane rings are in the energetically favourable chair conformation. However, a green blob can be seen in its PDB-style electron density map near one cyclopentane atom, suggesting that the atomic coordinates may need adjustment. Adjustment of the ring structure should involve a conformational change in the cyclopentane ring to better fit the electron density.

Moreover, the cyclopentane ligand is not substituted. It only has 3 interactions (two of them with water molecules). Its binding interactions can be seen here: https://www.ebi.ac.uk/pdbe/entry/pdb/4r91/bound/3KT. Therefore, there is no structural reason for its flat conformation.

#### Proper flat conformation of a cyclopentane in PDB entry 7zxz

The structure of PDB entry 7zxz [46], released in 2022, includes a ligand with CCD ID K9R. The structure was determined at a resolution of 1.45 Å.

**Fig. 8:**
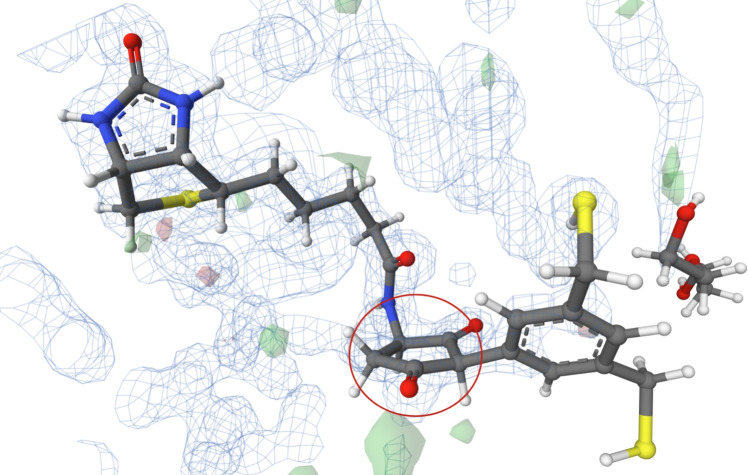
The ligand K9R (chain E, residue number 201) from PDB entry 7zxz with cyclopentane ring in flat conformation (highlighted in a red circle). The ligand is surrounded by electron density, which is visualised using a PDB-style electron density map [33]. The Mol* visualisation software [17] was used to prepare the figure

The structure model contains two K9R ligands, both of which have a ring in the planar conformation. We focused on the ligand in chain E (see Fig. 8). Although the entire structure of the ligand is not fully covered by electron density, the analysed ring is. There are no green or red blobs of electron density in the immediate proximity.

We can observe that this ring is not perfectly flat. One carbon atom is slightly out of the plane formed by the other four carbons, creating a hint of an envelope conformation. The analysed cyclopentane is held in this near-flat conformation by two double-bonded oxygen atoms and the spatial orientation of the bonded benzene ring. The experimental data, as well as the substituents of the ring, prove the correctness of the chosen conformation.

#### Poorly fitted chair conformation of a benzene in PDB entry 3ilz

The structure of PDB entry 3ilz [47] includes a ligand with CCD ID B72. This ligand has been proposed as a potential drug candidate for the treatment of hypercholesterolemia and obesity. The structure 3ilz was released in 2010 and has a high resolution of 1.85 Å. On the other hand, the validation report for this PDB entry indicates several quality issues of the structure model, such as a high clashscore [48, 49].

**Fig. 9:**
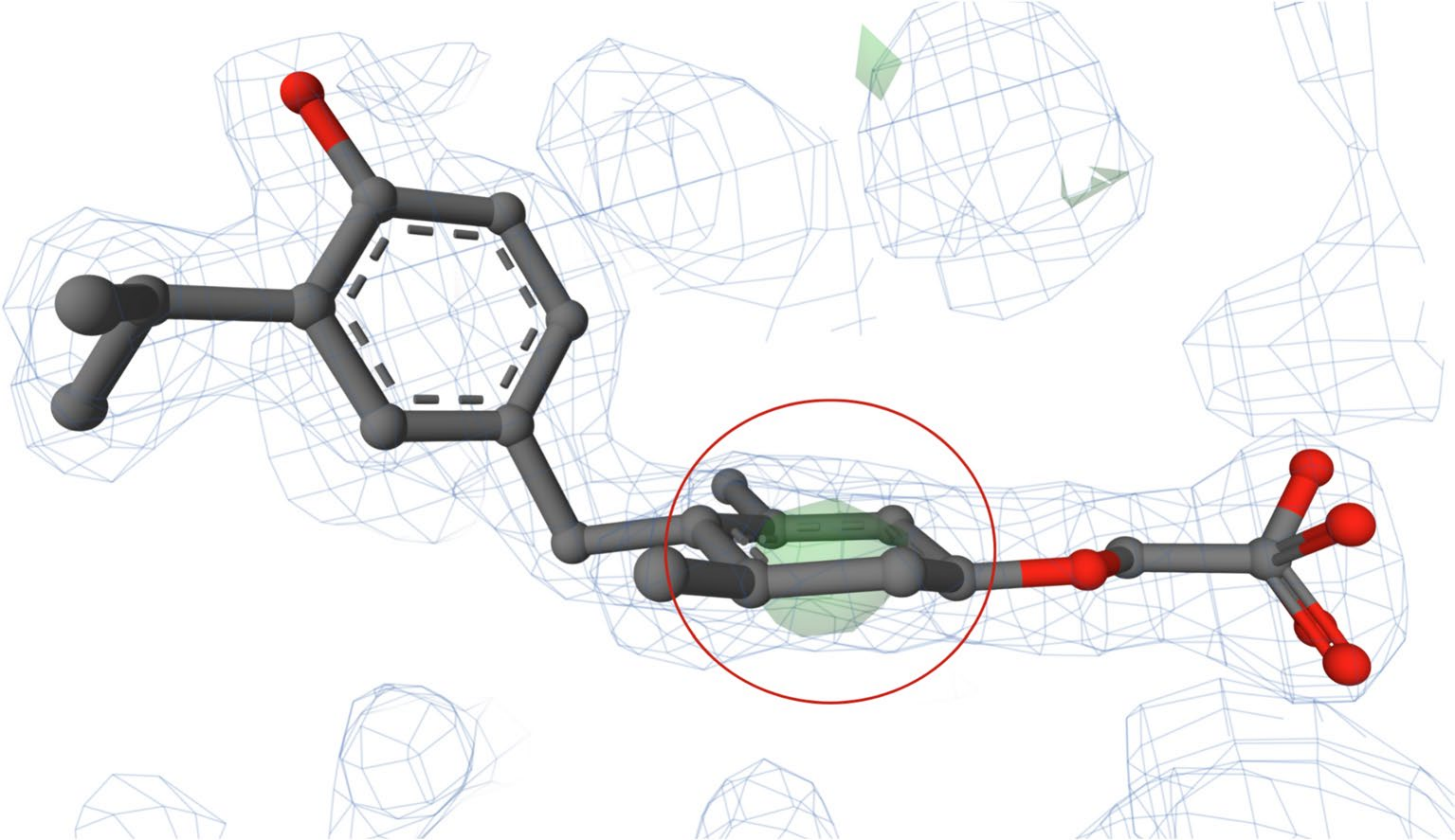
The ligand B72 (chain B, residue number 1) from PDB entry 3ilz [47] with a benzene ring in the chair conformation (highlighted in a red circle). The ligand is surrounded by electron density, which is visualised using a PDB-style electron density map [33]. The Mol* visualisation software [17] was used to prepare the figure

In the B72 ligand in this structure model, we found a benzene ring adopting a chair conformation (see Fig. 9). The whole ligand, including its benzene ring, is fully covered by electron density, as shown in the PDB-style electron density map. However, the electron density does not support the occurrence of benzene in the chair conformation. It is documented by the appearance of a green blob on the map (see Fig. 9). Since this chair conformation is not present in the corresponding PDB-REDO model, we attribute its occurrence to incorrect restraints during refinement.

## Conclusion

Our analysis reveals conformational behaviour of cyclopentane, cyclohexane, and benzene rings occurring in ligands, which are parts of experimental protein structures deposited in the PDB database. We found that the conformational behaviour strongly differs among these three ring types.

Cyclohexane is the most interesting ring from the conformational point of view. We found 5 852 instances of cyclohexane rings in the PDB, and more than 22 % of them (i.e., 1 676) were in energetically unfavourable conformations. A significant subset (i.e., 215) was supported by experimental data. We examined these structures and found that the occurrence of boat, half-chair, and twist-boat conformations has a chemical reasoning in particular ligands. On the other hand, the energetically unfavourable conformation is revealed as artificial in many structures, and the chair conformation would fit better with their experimental data. For flat cyclohexane structures, it seems all such occurrences are caused by a poor fit or an annotation error.

For cyclopentane, we found 5 100 occurrences in the PDB, and only about 5 % of rings (i.e., 264) were in an unfavourable conformation. From these structures, 36 were supported by sufficient experimental data. In contrast to cyclohexane, cyclopentane flat conformations are chemically reasonable for certain ligands (e.g., those containing multiple substituents). However, like cyclohexane, many energetically unfavourable cyclopentane structures result from poor electron density fit or other structure determination errors.

For benzene, we extracted 115 384 ring structures. Only 58 of them were not in the favourable flat conformation, and only 16 of them were supported by experimental data. All these 16 structures have model quality issues.

To summarise, energetically unfavourable ring structures can occur in cyclopentane and cyclohexane ligands for proper chemical reasons. Their examination can help us understand the binding of these ligands, which can be helpful for pharmacology or chemoinformatics. Still, energetically unfavourable ring conformations are often caused by model quality issues. Therefore, their occurrence should motivate researchers to inspect the quality of the experimental model of the protein and also the ring’s fit to the experimental data. Unfortunately, information about ring conformations is not currently available in the PDB.

For this reason, it would be useful to include data on conformations of common rings in PDB validation reports. These reports should also contain information about energetical favourability of ring conformations. If a researcher finds unfavourable conformations in structures of interest, they can use PDB-REDO to evaluate their relevance.

It would also be helpful to add information about ring conformations to the PDB deposition pipeline in the wwPDB OneDep system [50]. This would allow the structure author to see the occurrences of unfavourable conformations and correct them during the deposition process if necessary.

Our plan for the future is to provide an analysis of all common ring types in the PDB. In parallel, we plan to initiate discussions with the Protein Data Bank in Europe [51] about adding this information to PDB validation reports and the PDB deposition pipeline.

## Data Availability

All the software used in the workflow can be freely and openly accessed in a GitHub repository (https://github.com/sb-ncbr/rings-conformation-validation). The input data used for this analysis, the results, and a snapshot of the workflow, as well as optimised conformations for all three analysed ring types, a table with CCD entries sorted by the number of unfavourable conformations and CSV files containing assigned conformation, resolution, and electron density coverage of each analysed ring are freely and openly available in a persistent public share (https://doi.org/10.58074/6krq-v784) in the Onedata system for data management and storage. Additionally, the analysis for 1.2 $\sigma$ is included in the results archive. Please note that the complete companion dataset to this article consists of nearly 800 GB of data.

## Abbreviations

CCD: Chemical Component Dictionary
CSV: Comma-separated values
mmCIF: Macromolecular crystallographic information file
PDB: Protein Data Bank
